# PAX-5 positive anaplastic large cell lymphoma presenting by dysphagia; a case report 

**Published:** 2017

**Authors:** Negar Azarpira, Akbar Safaie, Ahmad Monabati, Masudd Hosseinzadeh, Sadat Noori, Maryam Moini, Alireza Rahimi, Tahereh Heidari, Marjan Rahsaz

**Affiliations:** 1 *Transplant Research Center, Shiraz University of Medical Science, Shiraz, Iran*; 2 *Pathology Department, Shiraz University of Medical Science, Shiraz, Iran*; 3 *Gastroenterology Research Center, Department of Internal Medicine, Shiraz University of Medical Science, Shiraz, Iran *

**Keywords:** PAX5, Anaplastic large cell lymphoma, Esophagus, stricture

## Abstract

Anaplastic large cell lymphoma (ALCL) is a distinct pathologic entity with characteristic morphologic, im¬munophenotypic and cytogenetic features. Obstructive symptoms are rare presentation of ALCL. We report a 16-year-old boy who initially presented with dysphagia. Upper gastrointestinal endoscopy revealed severe stenosis with an infiltrative process starting from 24 cm of incisors in lower esophagus Esophageal mucosal biopsy demonstrated lymphomatous involvement that ancillary tests confirmed the diagnosis of ALCL, ALK (kinase-positive), and PAX5 positive. The patient responded to CHOP-based chemotherapy. This case illustrated an unusual presentation of primary Non Hodgkin lymphoma of esophagus.

## Introduction

 Anaplastic large cell lymphoma (ALCL) is a distinct pathologic entity with characteristic morphologic, im¬munophenotypic and cytogenetic features. Obstructive symptoms are rare presentation of ALCL (1-4). We report a 16-year-old boy who initially presented with dysphagia. Upper gastrointestinal endoscopy revealed severe stenosis with an infiltrative process starting from 24 cm of incisors in lower esophagus Esophageal mucosal biopsy demonstrated lymphomatous involvement that ancillary tests confirmed the diagnosis of ALCL, ALK (kinase-positive), and PAX5 positive. The patient responded to CHOP-based chemotherapy. This case illustrated an unusual presentation of primary Non Hodgkin lymphoma of esophagus. 

## Case Report

A 16- year-old boy was referred to our hospital for progressive esophageal dysphagia over a period of one month. His complaint had been investigated in another medical center before his referral to our center and he had received some treatment without improvement. 

The patient’s past medical history was unremarkable. There was no previous history of corrosive ingestion or instrumentation of the esophagus. Physical examination did not reveal any specific abnormality. In review of systems he did not have any symptoms of fever, night sweats or weight loss (B symptoms).

Routine blood tests were normal. Human immunodeficiency virus (HIV) tests were negative.

The first upper endoscopy showed a severe stenosis from an infiltrative process starting from 24 cm of incisors in lower esophagus which made scope passage impossible. Tissue biopsies were taken that was inadequate for diagnosis. Spiral CT scan of the chest revealed significant dilatation of proximal of esophagus with narrowing at the level of carina with wall thickening. No lymphadenopathy or mediastinal widening was identified. In the next endoscopic session, the stenotic area was dilated using bougie dilators. Abnormal tissue infiltration was observed to involve the mid part of esophagus done to 30 cm from incisors. Stomach and duodenum were normal in endoscopic view.

**Figure 1:**
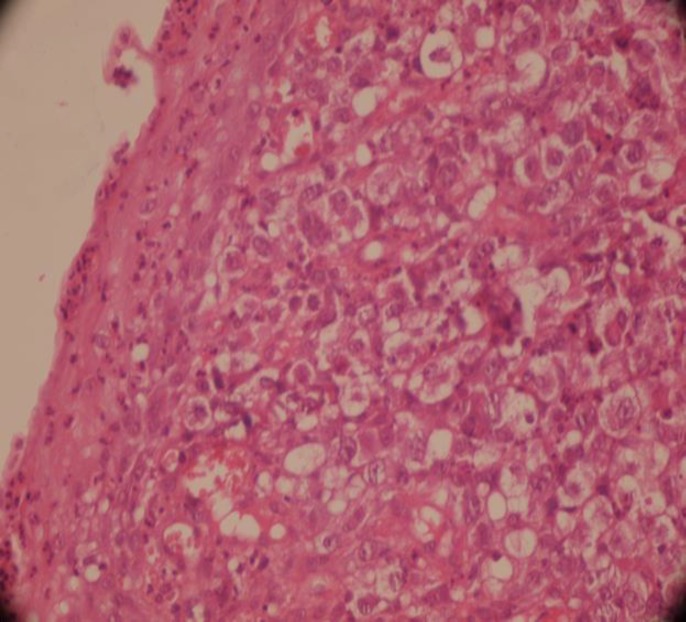
Squamous epithelial lining of esophagus. Medium-to-large sized neoplastic cells are infiltrated beneath the epithelium

The biopsy showed tiny fragments of soft tissue with squamous epithelial lining. Under the epithelium, sheets of large sized neoplastic cells, mixed with reactive inflammatory cells, were noticed. The large neoplastic cells had moderate amounts of eosinophilic cytoplasm, with single to several nucleoli. Occasional kidney shaped nuclei were also present. Classic Reed-Sternberg cells were absent (Figure 1).

**Figure 2:**
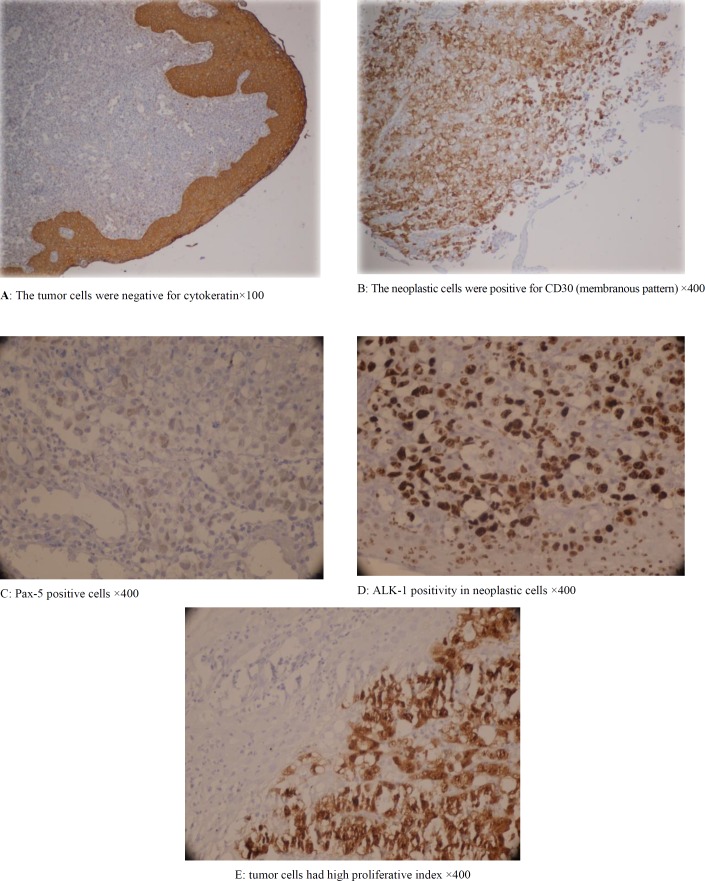
Immunohistochemical study

Immunohistochemistry revealed that the neoplastic cells were positive for CD45 and negative for CD3, CD5, CD7, CD4, and CD8. Pan B cell markers (CD20, CD79a), cytokeratin (Fig 2A), and the epithelial membrane antigen were all negative. CD30 was strongly positive (membranous pattern) (Fig 2B) and Pax-5 was expressed in tumor cells (Fig 2C). CD15 was only present in neutrophils. ALK-1(anaplastic lymphoma kinase) immunostain was positive (Fig 2D). Approximately, 65% of the tumor cells were positive for ki67 (Fig 2E). The overall findings were consistent with non-Hodgkin’s lymphoma, ALCL Alk+ (5). 

Unfortunately, the Endoscopic ultrasound (EUS) was not done. There were no lesions in the colonoscopy. Bone marrow biopsy was normal with no evidence of lymphomatous involvement. The patient received CHOP-based chemotherapy, with partial relief of the dysphagia. 

## Discussion

Gastrointestinal (GI) lymphoma is not uncommon, especially in the stomach and small intestine. In contrast, esophageal lymphomas are rare and comprise <1% of all GI lymphomas (6,7). In this setting, extension either from primary gastric lymphoma or mediastinal lymph nodes must be ruled out. To the best of our knowledge, twenty one cases of primary esophageal lymphoma were reported in the literature review (6,7).

The majority of primary esophageal lymphomas are composed of diffuse large B-cell and mucosa-associated lymphoid tissue lymphomas (MALTOMA) (6,7). Only a few cases of primary esophageal ALCL ALK positive were reported (8-13). 

HIV infection is considered as a major risk factor for developing hematopoietic malignancies especially for T-cell lymphoma and ALCL. The majority of the HIV-associated ALCL are ALK negative (13,14). Our English literature review identified seven cases with primary esophageal ALCL of esophagus (8-13) including the current case. 

There were 7 patients in total with 6 males and 1 female (male to female ratio of 6:1). The patients’ age was from 3 to 66 years. The major site of involvement was middle to lower part of the esophagus. No definite radiological finding was identified, and barium swallow studies resembled carcinoma. 

In endoscopy, different findings such as ulcerative masses, polypoidal lesions, and submucosal nodules were reported (8-13). It is rare for ALCL to initially present with dysphagia and stricture. In our patient, the lymphoma is considered primary because only the esophagus is involved. 

ALCL must be con¬sidered in differential diagnoses of carcinoma if esophageal obstruction is suspected, especially in young adults (10). Immunohistochemical studies on tissue biopsy are mandatory for distinguishing the lymphoma from carcinoma and for differentiating between B and T cell subtypes. The aberrant expression of PAX5 in the context of anaplastic large cell lymphomas should be interpreted cautiously. The major differential diagnosis is classical Hodgkin lymphoma. The intensity of staining in PAX5-positive lymphoma cell in ALCL is similar to that typically seen in Hodgkin cell (14).

Therefore, consideration of cytologic features of the tumor cells, cellular background, expression of B and T antigen as well as clinical features are necessary to make the appropriate diagnosis. In cases with questionable lineage, molecular studies are suggested (5).

As it has previously been mentioned, the presence of chromosomal translocations leads to expression of ALK fusion proteins and subdivision of this tumor into ALK+ and ALK- sub-classes. According to the ALK fusion partner, ALK staining in immunohistochemical study may be cytoplasmic, nuclear and nucleolar or, the cell membrane (4,15).

In 15–85% of systemic ALCLs ALK fusion pattern is t(2;5) (p23;q35) translocation, the ALK gene at 2p23 fuses with the nucleophosmin (NPM) gene at 5q35, which results in the fusion protein NPM–ALK. Less frequently, fusion of the ALK gene to a gene other than NPM may occur. Overall, the ALK protein expression is associated with a good prognosis (15,16). Distinction of ALK+ALCL from ALK–ALCL is clinically important. ALK+ ALCL usually presents with advanced stage with extranodal involvement, but is associated with more response to chemotherapy and better 5-year survival (16). ALK+ ALCL patients produce antibodies to ALK and show an immune response to the tumor cells. It seems that higher titer is associated with better prognosis (4). ALK+ is very sensitive to chemotherapy. Adult patients are generally treated with Anthracycline - containing regimens [CHOP (cyclophosphamide, doxorubicin, vincristine, prednisone), CHOEP (CHOP + etoposide) (4).

ALCL, ALK- are usually treated with similar chemotherapy protocols. The event-free survival rates of ALK+ disease is about 65-75%; however, in ALK- disease it ranges from 15% to 46% (4,16). New drugs such as Brentuximab vedotin (an anti-CD30 antibody) and ALK inhibitors are very promising treatments for ALCL patients (4,17).
